# Transplantation of Oligodendrocyte Precursor Cells Improves Locomotion Deficits in Rats with Spinal Cord Irradiation Injury

**DOI:** 10.1371/journal.pone.0057534

**Published:** 2013-02-27

**Authors:** Yan Sun, Chong-Chong Xu, Jin Li, Xi-Yin Guan, Lu Gao, Li-Xiang Ma, Rui-Xi Li, Yu-Wen Peng, Guo-Pei Zhu

**Affiliations:** 1 Department of Anatomy, Histology and Embryology, Shanghai Medical College, Fudan University, Shanghai, China; 2 Institutes of Biomedical Sciences, Fudan University, Shanghai, China; 3 Department of Radiation Oncology, Shanghai Cancer Hospital, Fudan University, Shanghai, China; Université Pierre et Marie Curie-Paris6, INSERM, CNRS, France

## Abstract

Demyelination contributes to the functional impairment of irradiation injured spinal cord. One potential therapeutic strategy involves replacing the myelin-forming cells. Here, we asked whether transplantation of Olig2^+^-GFP^+^-oligodendrocyte precursor cells (OPCs), which are derived from Olig2-GFP-mouse embryonic stem cells (mESCs), could enhance remyelination and functional recovery after spinal cord irradiation injury. We differentiated Olig2-GFP-mESCs into purified Olig2^+^-GFP^+^-OPCs and transplanted them into the rats’ cervical 4–5 dorsal spinal cord level at 4 months after irradiation injury. Eight weeks after transplantation, the Olig2^+^-GFP^+^-OPCs survived and integrated into the injured spinal cord. Immunofluorescence analysis showed that the grafted Olig2^+^-GFP^+^-OPCs primarily differentiated into adenomatous polyposis coli (APC^+^) oligodendrocytes (54.6±10.5%). The staining with luxol fast blue, hematoxylin & eosin (LFB/H&E) and electron microscopy demonstrated that the engrafted Olig2^+^-GFP^+^-OPCs attenuated the demyelination resulted from the irradiation. More importantly, the recovery of forelimb locomotor function was enhanced in animals receiving grafts of Olig2^+^-GFP^+^-OPCs. We concluded that OPC transplantation is a feasible therapy to repair the irradiated lesions in the central nervous system (CNS).

## Introduction

The spinal cord is one of the important dose-limiting normal tissues in clinical radiotherapy. Excessive doses of radiation to the spinal cord result in radiation injury, which is a rare but serious complication of radiotherapy for cancer [1], [2]. The pathophysiology involved in irradiation-induced spinal cord injury is demyelination caused by death of oligodendrocytes [3], [4]. Reduction in the number of oligodendrocytes was observed as early as 24 hours after X-ray irradiation [5], [6]. There is a persistent decline in the number of OPCs from two weeks to three months after X-ray irradiation [7], [8]. Remyelination often fails because of primary deficiency in the precursor cells, failure of precursor cells’ recruitment, or incompetence of differentiation and maturation of precursor cells [9]. Persistent demyelination may result in further axonal loss. Such changes could be associated with permanent motor and sensory deficits [10], which become fatal if the damage occurs at the upper cervical level [11]. There are currently no therapeutic approaches that promote remyelination available in a clinical setting.

Cell therapy offers an attractive option as grafted cells may replace the lost ones as well as provide neurotrophic benefits to surrounding tissue [9], [12]. To date, primary cultured adult neural stem cells, Schwann cells and olfactory ensheathing cells have been transplanted into the radiation-injured spinal cord [13], [14], [15], [16], [17]. However, these cells have limited capacity in producing oligodendrocytes, the primary cell type that is damaged in radiation injury. Oligodendrocyte precursor cells (OPCs), which can be isolated from brain tissues [18], [19], offer an alternative source. Nevertheless, they need to be derived from brain tissues. Embryonic stem cells (ESCs) may become a suitable candidate because they are genetically normal, pluripotent, and capable of indefinite replication, and they can be differentiated to all the cell types in the body, including OPCs [20], [21], [22]. Although the induced differentiation of ESCs is a well-developed approach, there are still problems, especially the purity of target cells [23]. Therefore, it is important to develop strategies for the directed differentiation of ESCs into specialized functional cell types and/or purification of the target cells *in vitro* before transplantation.

In the present study, we employed a mESCs line that carries a GFP reporter in the locus of Olig2, a transcription factor that is critical for OPCs development [24]. Following transplantation of Olig2^+^-GFP^+^-OPCs into the spinal cord of rat that underwent irradiation injury, the grafted OPCs survived, differentiated to myelinating oligodendrocytes and improved locomotor function of the injured rats.

## Materials and Methods

### Ethics Statement

This study was carried out in strict accordance with the recommendations in the Guide for the Care and Use of Laboratory Animals of the National Institutes of Health. The protocol was approved by the Committee on the Ethics of Animal Experiments of Fudan University (Permit Number: SCXK 2009-0019). All efforts were made to minimize suffering.

### Irradiation Injury of the Spinal Cord

Female Wistar adult rats (180–200 g, Slrc Laboratory Animal, China) were anesthetized by an intraperitoneal injection of 7.5 mg/kg ketamine, 60 mg/kg xylazine and immobilized during irradiation. The rats’ cervical spinal cords were irradiated using a 6 Mev Electron Beam Linear Accelerator (Elekta AB, Stockholm, Sweden). A 22 Gy radiation dose was delivered to a 2 cm×2 cm radiation field, maintaining cervical 4–5 as the center of the electron-irradiated zone. Radiographs were taken before and after X-irradiation to confirm that all the animals were in the same position. After irradiation, the animals were given subcutaneous saline (5 ml) and housed in a 25°C warm room.

### Derivation of Olig2^+^-GFP^+^-OPCs from Olig2-GFP-mESCs

Olig2-GFP-mESCs were the gift of Dr. Su-Chun Zhang (University of Wisconsin, USA) and have been previously described [25]. Olig2-GFP-mESCs were differentiated according to a modified protocol [25]. Briefly, mESCs were trypsinized and placed onto low attachment flasks (Greiner, Germany) in a neural differentiation medium containing DMEM/F12, N2 supplement, leukemia inhibitory factor (LIF), nonessential amino acids, L-glutamine, 2-mercaptoethanol and 10% knockout serum replacement (all from Invitrogen, Carlsbad, CA, USA). Under this condition for 2 days, mESCs formed small aggregates. Retinoic acid (RA, 0.5 µM, Sigma, St Louis, MO, USA) and Purmorphamine (Pur, 0.5 µM, Calbiochem, San Diego, CA, USA) were added at days 2–6 to induce neural progenitors. The aggregates were cultured in suspension in the neural differentiation medium with bFGF (20 ng/ml, R&D systems) and heparin (2 µg/ml, Sigma) for another 6 days. On day 12, the differentiated aggregates were dissociated with trypsin-EDTA (0.05%, Gibco) and plated onto flasks coated with Matrigel in modified Bottenstein-Sato medium. The modified Bottenstein-Sato medium contained insulin (10 µg/ml), BSA (100 µg/ml), human transferrin (100 µg/ml), progesterone (60 µg/ml), sodium selenite (40 µg/ml), N-acetyl-cysteine (60 µg/ml), putrescine (16 µg/ml), biotin (10 ng/ml) and cAMP (5 µM) (all reagents from Sigma). T3 (40 ng/ml, Sigma), PDGF-AA (10 ng/ml) and NT3 (5 ng/ml) (all from R&D systems) were added to promote the proliferation of the OPCs.

For immunofluorescence, dissociated spheres (2×10^4^ cells/µl) were plated on coverslips coated with poly-L-lysine and human laminin (Sigma).

For fluorescence activated cell sorting (FACS), cells were harvested at day 12, gently dissociated to single cells and washed with a FACS buffer (PBS, 1%N2, 200 mM L-glutamine, 55 mM β-ME, 50 ng/ml NAC). Cells were analyzed by a Becton Dickinson FACSCaliber with CellQuest Pro (BD Biosciences, San Diego, CA, USA).

For transplantation, FACS-sorted Olig2^+^-GFP^+^-OPCs were resuspended in a fresh culture medium and cultured for 2 days. At day 14, cells were dissociated with accutase (Invitrogen) and prepared at approximately 100,000 cells/µl in artificial cerebrospinal fluid (aCSF). Trypan blue exclusion testing indicated that this preparation consisted of 95% viable cells at time of transplantation.

### Cell Transplantation

Transplantation was performed as described [26]. The animals were immunosuppressed using cyclosporine A (10 mg/kg/d, s.c; Novartis Pharma Schweiz AG, Switzerland) 1 day prior to transplantation and then every day for the duration of the study. Cell transplantation occurred 4 months after irradiation surgery. The animals were anesthetized as above, and the spinal cord was exposed by laminectomy at the cervical 4–5 vertebral level. After immobilization of the spinal cord, a 10 µl Hamilton syringe (Hamilton, Reno, NV) was lowered into the spinal cord using a stereotactic manipulator arm. Cell suspension (2 µl) was injected into two sites along the midline at a depth of 1.2 mm, 4 mm cranial and 4 mm caudal to the lesion epicenter, at a rate of 1.0 µl/min. The needle was removed after 5 min. The animals in which the injected solution was observed to exit from the needle track during injection or after withdrawal of the needle were omitted from the experiment. Animals receiving the same surgery and injection of 2 µl aCSF solution (without cells) were served as controls.

### Histology

For the histological analysis, the animals were deeply anesthetized with an overdose of 3% pentobarbitone and perfused with 0.9% saline following by 4% ice-cold paraformaldehyde prepared with phosphate buffer (0.1 M, pH7.4). The spinal cord was divided into eight 1 mm blocks that extended 4 mm cranial to and 4 mm caudal to the injury epicenter. Alternate blocks were processed to produce cryostat sections. Some of the cryostat sections were stained with LFB/H&E to examine general morphology and the extent of demyelination. Some of the cryostat sections were used for immunofluorescent analysis of grafted cells.

To quantify the demyelination of injured spinal cord, we used Image J (http://rsbweb.nih.gov/ij/index.html) to measure the relative optical density of the dorsal funiculus from all groups. A pre-defined region of interest was selected to encompass the dorsal funiculus. Color images were acquired at the same exposure level, coverted to 8-bit gray scale, and the mean density calculated from the threshold pixels excluding the background. Density was analyzed in four regions of the dorsal funiculus per section. Eight sections per spinal cord were analyzed. There were six animals in each group.

### Immunofluorescence

Immunostaining was performed on cultured cells or cryosectioned spinal cord using standard protocols [27]. Primary antibodies used in present study included mouse anti-Oct4 (1∶2000, Santa Cruz Biotechnology, Santa Cruz, CA), goat anti-Sox2 (1∶1000, R&D Systems), rabbit anti-NG2 (1∶500, Chemicon International Inc, Temecula, CA, USA), rabbit anti-O4 (1∶50, Santa Cruz), rabbit anti-PDGFRα (1∶500, Santa Cruz), rabbit anti-MBP (1∶500, Abcam, Cambridge, MA), goat anti-GFP (1∶200; Abcam), mouse anti-APC (1∶1000; Abcam), mouse anti-p75 (1∶1000; Chemicon), rabbit anti-GFAP (1∶500; Chemicon), and mouse anti-NF (1∶2000; Chemicon). Coverslip cultures or spinal cord sections were incubated in a blocking buffer (10% donkey serum and 0.2% Triton X-100 in phosphate buffer saline) for 60 min at room temperature before being incubated in the primary antibodies overnight at 4°C. Fluorescently conjugated secondary antibodies were used to reveal the binding of primary antibodies (1∶2000, Invitrogen) and nuclei were stained with 4,6-dianidina-2-phenylindole (DAPI) (0.1 µg/ml, Sigma). The immunofluorescent samples were visualized using a Nikon-Eclipse TE 2000-S fluorescence microscope (Nikon Instruments, Sterling Heights, MI, USA) or a Leica TCS-SP5 laser-scanning confocal microscope (Leica, Germany).

To quantify the differentiation pattern *in vitro*, the percentage of immunopositive cells was determined by dividing the total number of immunopositive cells by the total number of GFP/DAPI positive cells in each imaging field and then averaging the result from three fields per marker. These fixed fields were randomly selected from biological replicates using “Image J”.

To assess the differentiation of grafted Olig2^+^-GFP^+^-OPCs in the injured spinal cord, four coronal sections, 200 µm apart, were taken spanning the lesion epicenter. The percentage of engrafted OPCs that co-expressed NG2, APC, MBP, p75 and GFAP was quantified for the grafted animals.

### Electron Microscopy

For electron microscopic processing, the animals were sacrificed by aortic perfusion with isotonic, heparinized saline followed with 4% paraformaldehyde and 1% glutaraldehyde in 0.1 M phosphate buffer. The fixed spinal cords were washed in 0.1 M phosphate buffer and embedded in 3% agar. Transverse sections (40 µm thick) were obtained on a vibrotome (Leica, VT1000S, Nussloch, Germany) and mounted onto slides. The sections were washed in PB and subjected to an additional fixation in 2% OsO_4_ in phosphate buffer for 2 h at room temperature. After sequential dehydration in increasing concentrations of ethanol (50–100%) and pure acetone, the sections were embedded in Epon 812. Ultrathin sections (80 nm) obtained using an ultramicrotome (UI-tracut E, Reicher-Jung, NY, USA) were stained with urany1 acetate and lead citrate, examined and photographed under a transmission electron microscope (Philips CM120, Amsterdam Netherlands).

### Behavioral Assessments

The clinical degree of weakness was graded according to a scale developed by Ushio et al. [28], [29]: grade 0 = normal; grade 1 = forelimb instability seen only when the animal jumps or runs; grade 2 = mild weakness, but able to run; grade 3 = moderate weakness, able to walk but not run; grade 4 = marked weakness, attempts to walk; grade 5 = severe weakness, purposeless movements of legs or complete paraplegia. All rats were allowed to live for 8 weeks after transplantation.

### Statistical Analysis

All data are reported as Mean±SEM. Repeated measures ANOVA followed by the Tukey-Kramer test was used for comparison of clinical grade scores at each time point. The difference between the two was compared using Student’s tests. Statistical analysis was performed using SPSS 16.0. Differences were considered to be statistically significant at *p*<0.05.

## Results

### Demyelination Induced by Irradiation

**Figure 1:**
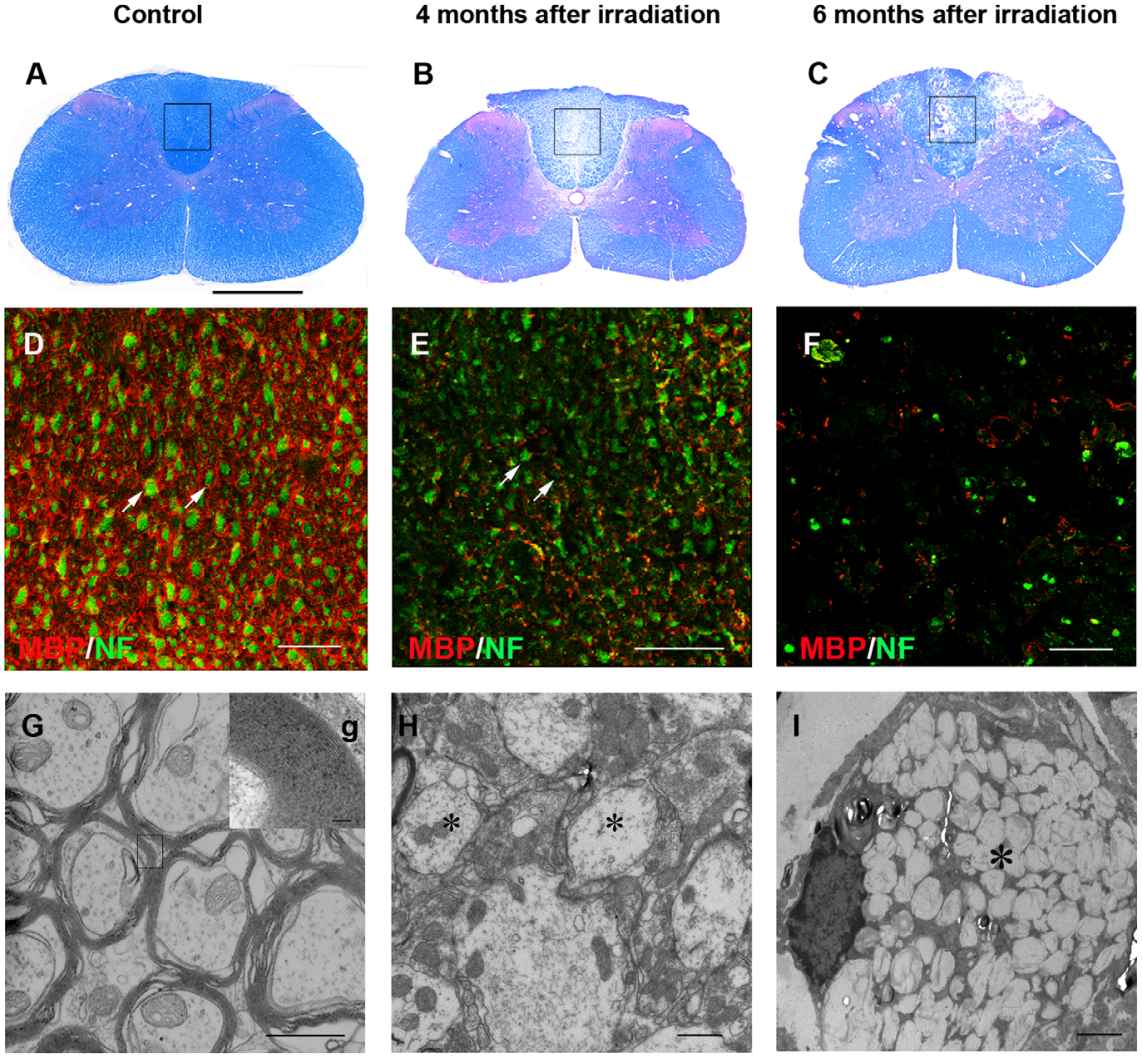
Radiation-induced demyelination in the dorsal funiculus of cervical spinal cord. (A) LFB/H&E staining showed normal spinal cord without irradiation. (B) Four months after irradiation, there was a focal demyelinated zone in the dorsal funiculus. (C) Six months following injury, focal necrosis was seen in the dorsal funiculus. (D) Immunostaining for MBP and NF, enlargement of framed area in A, depicted that non-irradiated myelin surrounding the axons (arrows). (E) Four months after irradiation, most of the axons lost myelin (arrows). (F) At six months, axons began to show necrosis. (G) Electron microscopy confirmed the normal structure of myelin. *g*, Higher magnification of the tissue in the box in G, indicated the compact myelin sheath. (H) Four months after irradiation, most of axons remained demyelinated (asterisk). (I) Six months following damage, axons necrosis was found (asterisk). Bar, 200 µm (A–C); bar, 50 µm (D–F); bar, 1 µm (G, H); bar, 100 nm (g); bar, 2 µm (I).

Non-irradiated animals (n = 6) did not show any evidence of demyelination (Fig. 1A, D and G). Even though there was no histological evidence of demyelination observed from 1 to 3-month after irradiation, 80% of the animals (n = 60) developed a focally demyelinated zone in the dorsal funiculus from the fourth month, as shown by LFB/H&E staining (Fig. 1B). This was confirmed by immunostaining for myelin basic protein (MBP) and neurofilment (NF), showing the presence of many axons but sharply reduced MBP staining (Fig. 1E). Electron microscopy showed many viable demyelinated axons in the dorsal funiculus (Fig. 1H). Six months after irradiation, focal areas of necrosis were apparent in the white matter (Fig. 1C), with MBP loss and axonal degeneration (Fig. 1F). Axon necrosis was also found by electron microscopy (Fig. 1I).

The clinical degree of weakness was consistent with the histological changes. The mean interval between irradiation and manifestation of the first neurological symptoms (grade 1 = forelimb instability seen only when the animal jumps or runs) was 112±14 days. Four months after irradiation, the animals (n = 12) that did not manifest neurological symptoms (grade 0 = normal) were omitted from the experiment.

### Generation and Purification of Olig2^+^-GFP^+^-OPCs from mESCs

**Figure 2:**
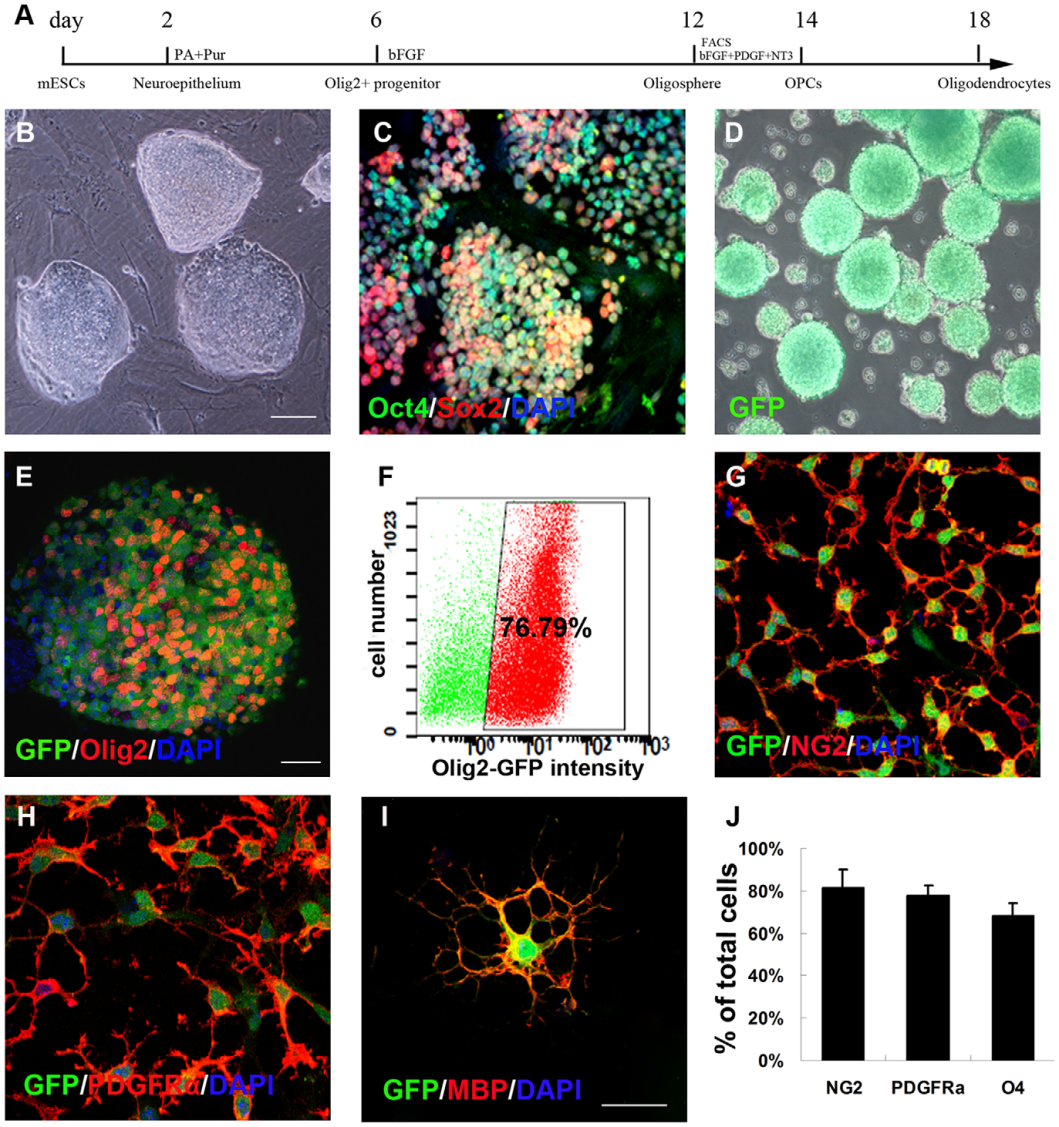
*In vitro* differentiation of Olig2-GFP-mouse mESCs into Olig2^+^-GFP^+^- OPCs. (A) Schematic procedure for differentiation of Olig2-GFP-mESCs into Olig2^+^-GFP^+^-OPCs. (B) Phase contrast photograph of mESCs colonies on mouse embryonic fibroblast. (C) mESCs remained undifferentiated and stained positive for Oct4/Sox2. (D) Phase contrast photograph of Olig2^+^-GFP^+^ spheres at day 12. (E) GFP^+^ spheres expressed Olig2. (F) At day 12, the percentage of GFP^+^ cells was 76.79%±1.35%, sorted by FACS method. At day 14, all purified OPCs expressed GFP as well as the OPC markers NG2 (G) and PDGFRα (H). At day 18, plated cells adopted a typical oligodendrocyte morphology characterized by multiple branches and expressed GFP and MBP (I). (J) Quantification of immunostaining: 81.69±8.16% of cells expressed NG2, 78±4.31% of cells expressed PDGFRα, and 68.47±5.59% of cells expressed O4. Data in J are expressed as the mean±SEM, n = 3 independent experiments, 6 total replicates. Bar, 200 µm (B–D); bar, 50 µm (E–H); bar, 30 µm (I).

We used an established mESCs line (Olig2-GFP-mESCs) in which GFP was knocked into the locus of Olig2, a transcription factor expressed by ventral spinal cord progenitors [24]. In order to identify and purify OPCs, we used a modified protocol established in the Zhang Lab to differentiate mESCs into OPCs [25] (Fig. 2A). The Olig2-GFP-mESCs, expanding as discrete colonies on mouse embryonic fibroblast (Fig. 2B) and expressing Oct4/Sox2 (Fig. 2C), were detached from mouse embryonic fibroblasts to initiate differentiation and grown as free floating embryoid bodies (EB) for 2 days in the medium before being treated with RA (0.5 µM) and Pur (0.5 µM). At day 6, GFP^+^ spheres began to appear and by day 12 almost all the spheres were green (Fig. 2D). FACS analysis, confirmed by confocal microscopy, indicated that 76.79± 1.35% of the total differentiated cells expressed Olig2 and GFP (Fig. 2E, F). To further purify the OPCs, we used FACS based on GFP expression. FACS isolated cells were plated in the presence of FGF2, PDGF-AA and NT3. The majority of GFP cells expressed NG2 (81.69±8.16%) (Fig. 2G, J), PDGFRα (78±4.31%) (Fig. 2H, J) and O4 (68.47±5.59%) (Fig. 2J). Further differentiation of the OPCs for 6 days resulted in generation of highly branched oligodendrocytes, as indicated by positive staining for MBP (Fig. 2I).

### Survival and Migration of Transplanted OPCs within the Spinal Cord

**Figure 3:**
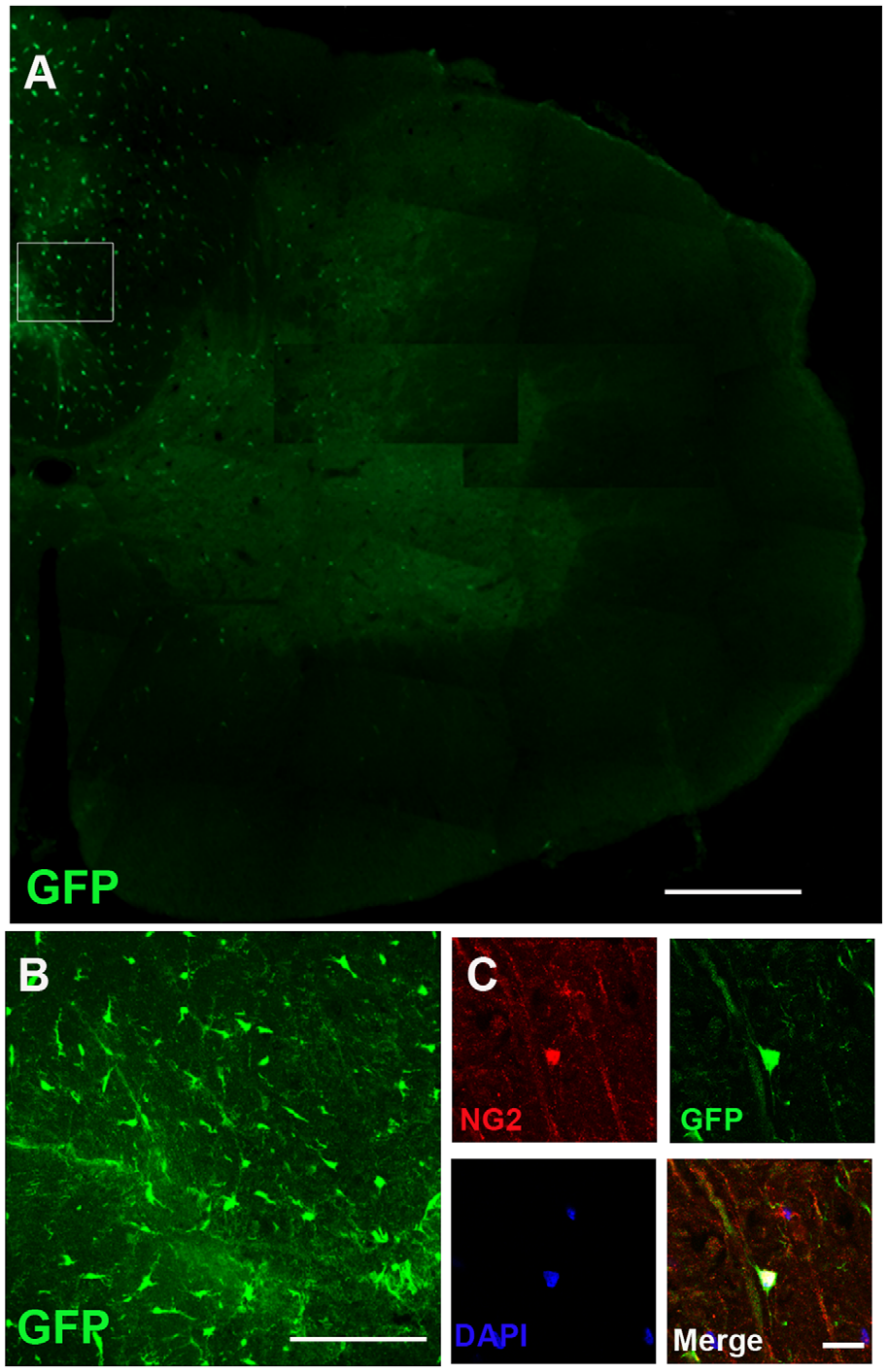
Distribution and morphology of Olig2^+^-GFP^+^-OPCs in irradiated spinal cord at four weeks after transplantation. (A) GFP^+^ cells were observed and distributed in the damaged spinal cord. (B) At higher magnification of the boxed area from (A), the GFP^+^ cells exhibited typical bipolar OPC morphology. (C) Many grafted Olig2^+^-GFP^+^-OPCs co-expressed the OPCs marker NG2. Bar, 250 µm (A); bar, 100 µm (B); bar, 20 µm (C).

A total of 24 injured animals received transplantation of Olig2^+^-GFP^+^-OPCs 4 months after irradiation. Four weeks after transplantation, grafted cells revealed by GFP, were found in the spinal cord of all transplanted animals. The animals (n = 24) without cell transplantation did not exhibit such labeling. The GFP-expressing cells were mainly distributed in the dorsal funiculus with a small number of cells migrating into the gray matter (Fig. 3A). At a higher magnification, the GFP^+^ cells adopted a typical OPCs morphology, as characterized by bipolar branches (Fig. 3B), and expressed the OPCs marker NG2 (which marks undifferentiated OPCs) (Fig. 3C), indicating that these grafted cells retain the OPCs identity.

### Transplanted OPCs Primarily Differentiate into Oligodendrocytes

**Figure 4:**
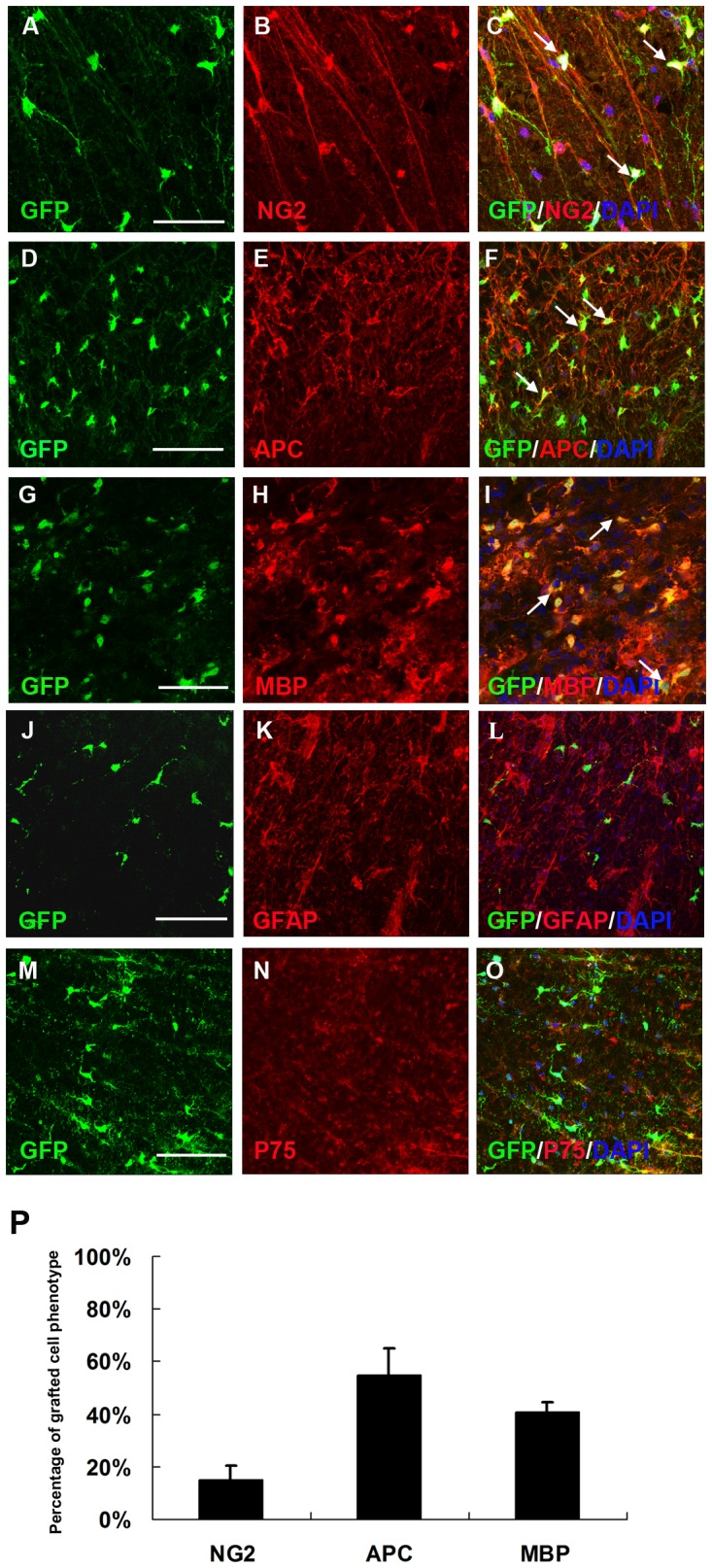
Olig2^+^-GFP^+^-OPCs primarily differentiate along the oligodendrocyte lineage. At eight weeks after transplantation, a certain number of grafted GFP^+^ cells still expressed NG2 (A–C, arrows) and most of the grafted GFP^+^ cells became APC-positive mature oligodendrocytes (D–F, arrows). Double staining for GFP and MBP in cross-sections further confirmed that GFP-immunoreactive rings were composed of MBP^+^ myelin (G–I, arrows). The grafted GFP^+^ cells did not co-expressed GFAP (J–L) or P75 (M–O). (P) Quantification of GFP^+^ cell populations in the spinal cord. Data are expressed as the mean number of cells/spinal cord±SEM (n = 6). GFP^+^ cells expressed NG2 (15.1±5.53%), APC (54.6±10.5%) and MBP (40.5±3.8%). Bar, 50 µm (A–C); bar, 35 µm (D–O).

Eight weeks after transplantation, 15.1±5.53% of the GFP positive cells were double labeled with NG2, a specific protein for precursor cells (Fig. 4A–C, arrows). Around 54.6±10.5% of GFP^+^ cells expressed APC (Fig. 4D–F, arrows), indicating that the majority of grafted OPCs differentiated into mature oligodendrocytes. About 40.5±3.8% of the grafted cells also expressed MBP, a major constitute of myelin (Fig. 4G–I, arrows). Although several previous studies [30], [31] showed that adult OPCs differentiated into Schwann cells after transplantation into the chemically demyelinated spinal cord, our transplanted OPCs did not express p75 (Fig. 4M–O). Other studies suggest that the microenvironment in the injured spinal cord induces grafted neural stem cells to differentiate primarily into astrocytes [14], [32]. In the present study, the transplanted OPCs did not differentiate into astrocytes marked by GFAP (Fig. 4J–L). In summation, the staining results revealed that Olig2^+^-GFP^+^-OPCs mostly differentiate into mature oligodendrocytes in the injured spinal cord. Over the course of the experiment, tumors, teratomas, or non-neuronal tissue formation were not observed in the transplant recipients.

### OPCs Transplantation Attenuated Demyelination after Radiation

**Figure 5:**
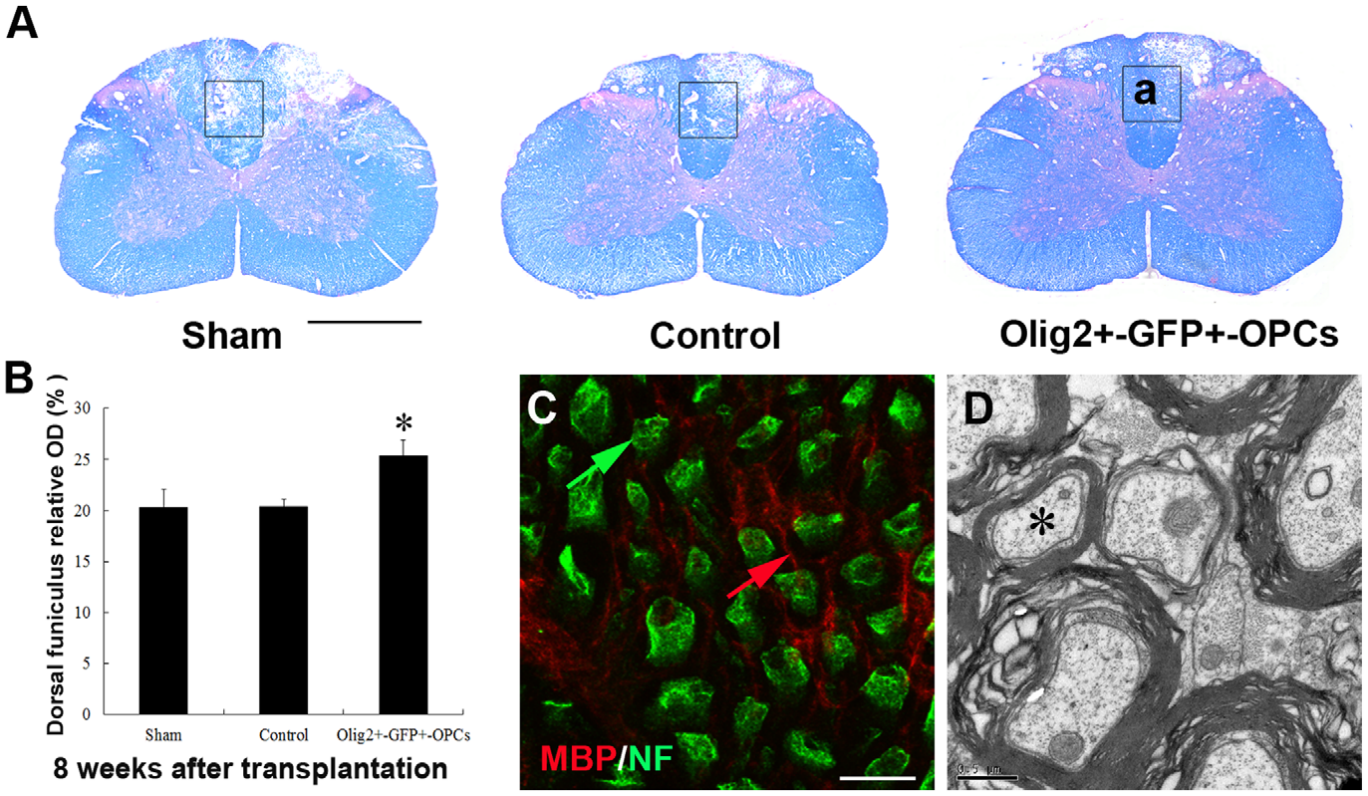
Transplantation of Olig2^+^-GFP^+^-OPCs results in a delayed demyelination and cavitation. (A) LFB/H&E staining showed smaller cystic cavitations in the transplanted cord than those in the sham and the control group in the dorsal funiculus. (B) Optical densities of dorsal funiculus in spinal cord was significantly increased in rats grafted with Olig2^+^-GFP^+^-OPCs, as compared to the sham and the control group (*p < 0.05, n = 12). (C) Enlargement of framed area in part a, illustrated immunostaining of MBP and NF, showing axons (green arrow) with or without myelin (red arrow). (D) Electron microscopy confirmed the structure of axons (asterisk). Bar, 200 µm (A); bar, 35 µm (C); bar, 0.5 µm (D).

Eight weeks after transplantation, irradiated spinal cord exhibited obvious demyelination and cavitation (Fig. 5A). However, the degree of demyelination was significantly decreased by OPCs transplantation as compared to the sham and the control group (Fig. 5B). Immunostainning for MBP and NF indicated numerous clusters of axons that maintained integrity (Fig. 5C). Electron microscopic analysis showed myelinated axons (Fig. 5D).

### OPCs Grafts Alleviated the Deterioration of Neurologic Function Following Injury

**Figure 6:**
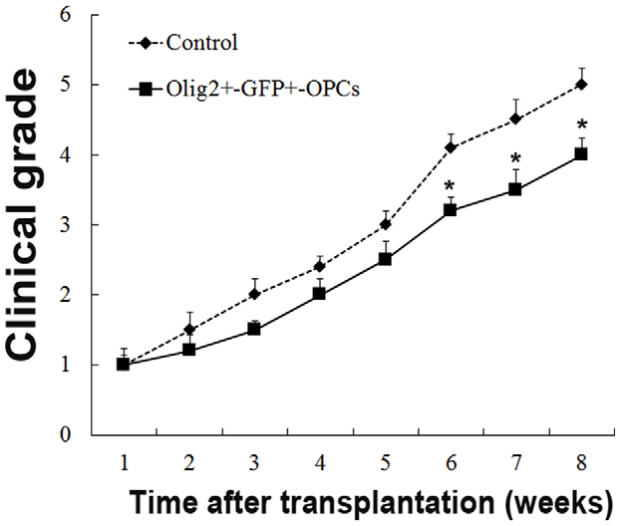
Forelimb locomotion is improved after transplantation of Olig2^+^-GFP^+^-OPCs. Locomotion function, as defined using the clinical grade score, was significantly reduced in Olig2^+^-GFP^+^-OPCs grafted animals at six to eight weeks after transplantation, as compared to the control group (*p < 0.05, n = 12).

To determine whether transplantation of OPCs improved recovery of function after injury, locomotion was assessed by using the clinical scoring. Six to eight weeks after transplantation, Olig2^+^-GFP^+^-OPCs grafted animals exhibited significantly lower clinical scores as compared to the control group (Fig. 6) (all *p*<0.05). The behavioral results showed that transplantation of Olig2^+^-GFP^+^-OPCs significantly improved forelimb functions.

## Discussion

In this study, we have directed mESCs to a purified population of OPCs based on the GFP expression in the Olig2 locus, which differentiate into oligodendrocytes *in vitro* and *in vivo*. More importantly, the oligodendrocytes derived from the Olig2^+^-GFP^+^-OPCs express MBP, an essential component of myelin sheath, which corresponds to correction of locomotion deficits in rats with irradiation injury of spinal cord. These results raise the prospect of cell-based therapy as a potential treatment for irradiation spinal cord injury.

Cell transplantation has been attempted for the radiation injured spinal cord. Adult rat neural stem cells differentiate predominantly into Schwann-like cells and generated peripheral myelin after transplantation into the irradiation injured spinal cord [14]. Olfactory ensheathing cells appear to migrate within X-irradiated area, they behave similarly as Schwann cells, which survive poorly and are unable to migrate significant distances when transplanted into either intact or X-irradiated spinal cords [16], [33]. OPCs are extremely motile and can differentiate into oligodendrocytes and produce myelin [34], [35]. Differentiation of oligodendrocytes from mESCs was explored by Billon and colleagues [36]. One of the greatest challenges facing ESCs research is the derivation of high purity target cells from pluripotent ESCs [23]. In the present study, we employed a mESCs line that carries a GFP reporter in the Olig2 locus. The modified differentiation protocol that we used is unique in that it generates highly enriched OPCs. FACS sorting resulted in an enriched population of OPCs (76.79±1.35%) (Fig. 2F). This provided us with a novel tool to investigate the ability of these cells to remyelinate demyelinated regions of irradiation spinal cord injury and determine whether remyelination confers functional benefit.

Most irradiation injury models are made using 40 Gy of X-irradiation which creates acute demyelination in the spinal cord [13], [14], [16], [37], [38]. However, the latent period of spinal cord irradiation in human has been reported to vary from 4 months to 4 years after cervical or thoracic irradiation [39]. In experimental animals, irradiation of the spinal cord leads to white matter necrosis within 3–8 months [3]. In the present study, we have used the dosage of 22 Gy to mimic the delayed demyelination of the spinal cord irradiation injury. The demyelination latent period was about 112±14 days, while the white matter necrosis and paralysis that occurred within 6 months of radiation exposure. Therefore, our model is similar to the clinical pathology changes of spinal cord irradiation injury.

Promoting remyelination is an important strategy to treat spinal cord injury. Chronic demyelination predisposes axons to degeneration [40], an irreversible event that is thought to be the major cause of progressive functional decline [41]. Previous studies observed cells with the capacity to myelinate following spinal cord injury [32], [34], [35], [42], [43], [44], [45]. The functional behavioral improvement is correlated with the capacity of the transplanted cells to integrate, differentiate, and myelinate axons at sufficient number [9], [46], [47]. In our study, we have found that transplanted cells migrate to the injured region and approximately 40% of them differentiate into mature oligodendrocytes expressing MBP. More importantly, immunostaining and electron microscopy analysis reveal the presence of many axons in the areas with Olig2^+^-GFP^+^-OPCs transplantation, suggesting the protection of axonal integrity by transplanted cells. Further study is needed by immunoelectron microscopy to prove remyelination occurred by transplanted cells.

The increased recovery in the transplantation group may also be explained by mechanisms other than remyelination. CNS neurons require multiple signals for optical survival and maturation, and continued oligodendrocyte-derived signals are necessary to maintain neuronal integrity [48]. In addition to their role in myelinating axons, oligodendrocytes release soluble factors, including insulin-like growth factor (IGF-1), glial-derived neurotrophic factor (GDNF) and brain-derived neurotrophic factor (BDNF), that promote neuronal survival, maintain axonal structure, and support synaptic plasticity in surviving axons [49], [50], [51], [52]. The expression and release of trophic factors offer a platform by which oligodendrocytes interact with neurons to form and maintain functional neural circuits in the injured spinal cord. Therefore, neural protection may be another mechanism by which grafted OPCs promote functional recovery.

## References

[1] Fowler JF, Bentzen SM, Bond SJ, Ang KK, van der Kogel AJ, et al (2000) Clinical radiation doses for spinal cord: the 1998 international questionnaire. Radiother Oncol 55: 295–300. 10869744 10.1016/s0167-8140(99)00133-4

[2] Bijl HP, van Luijk P, Coppes RP, Schippers JM, Konings AW, et al (2003) Unexpected changes of rat cervical spinal cord tolerance caused by inhomogeneous dose distributions. Int J Radiat Oncol Biol Phys 57: 274–281. 12909243 10.1016/s0360-3016(03)00529-7

[3] Okada S, Okeda R (2001) Pathology of radiation myelopathy. Neuropathology 21: 247–265. 11837531 10.1046/j.1440-1789.2001.00408.x

[4] Wong CS, Van der Kogel AJ (2004) Mechanisms of radiation injury to the central nervous system: implications for neuroprotection. Mol Interv 4: 273–284. 15471910 10.1124/mi.4.5.7

[5] Li YQ, Jay V, Wong CS (1996) Oligodendrocytes in the adult rat spinal cord undergo radiation-induced apoptosis. Cancer Res 56: 5417–5422. 8968095

[6] Atkinson S, Li YQ, Wong CS (2003) Changes in oligodendrocytes and myelin gene expression after radiation in the rodent spinal cord. Int J Radiat Oncol Biol Phys 57: 1093–1100. 14575841 10.1016/s0360-3016(03)00735-1

[7] Chari DM, Huang WL, Blakemore WF (2003) Dysfunctional oligodendrocyte progenitor cell (OPC) populations may inhibit repopulation of OPC depleted tissue. Journal of Neuroscience Research 73: 787–793. 12949904 10.1002/jnr.10700

[8] Hopewell JW, van der Kogel AJ (1999) Pathophysiological mechanisms leading to the development of late radiation-induced damage to the central nervous system. Front Radiat Ther Oncol 33: 265–275. 10549496 10.1159/000061239

[9] Franklin RJ, Ffrench-Constant C (2008) Remyelination in the CNS: from biology to therapy. Nat Rev Neurosci 9: 839–855. 18931697 10.1038/nrn2480

[10] Schultheiss TE, Kun LE, Ang KK, Stephens LC (1995) Radiation response of the central nervous system. Int J Radiat Oncol Biol Phys 31: 1093–1112. 7677836 10.1016/0360-3016(94)00655-5

[11] Schultheiss TE, Stephens LC, Peters LJ (1986) Survival in radiation myelopathy. Int J Radiat Oncol Biol Phys 12: 1765–1769. 3759528 10.1016/0360-3016(86)90317-2

[12] Franklin RJ, Kotter MR (2008) The biology of CNS remyelination: the key to therapeutic advances. J Neurol 255 Suppl 119–25. 10.1007/s00415-008-1004-6 18317673

[13] Rezvani M, Birds DA, Hodges H, Hopewell JW, Milledew K, et al (2001) Modification of radiation myelopathy by the transplantation of neural stem cells in the rat. Radiat Res 156: 408–412. 11554852 10.1667/0033-7587(2001)156[0408:mormbt]2.0.co;2

[14] Mothe AJ, Tator CH (2008) Transplanted neural stem/progenitor cells generate myelinating oligodendrocytes and Schwann cells in spinal cord demyelination and dysmyelination. Exp Neurol 213: 176–190. 18586031 10.1016/j.expneurol.2008.05.024

[15] Chari DM, Gilson JM, Franklin RJM, Blakemore WF (2006) Oligodendrocyte progenitor cell (OPC) transplantation is unlikely to offer a means of preventing X-irradiation induced damage in the CNS. Experimental Neurology 198: 145–153. 16410004 10.1016/j.expneurol.2005.11.023

[16] Lankford KL, Sasaki M, Radtke C, Kocsis JD (2008) Olfactory ensheathing cells exhibit unique migratory, phagocytic, and myelinating properties in the X-irradiated spinal cord not shared by Schwann cells. Glia 56: 1664–1678. 18551623 10.1002/glia.20718

[17] Monje ML, Mizumatsu S, Fike JR, Palmer TD (2002) Irradiation induces neural precursor-cell dysfunction. Nat Med 8: 955–962. 12161748 10.1038/nm749

[18] Zhang SC, Lundberg C, Lipsitz D, O’Connor LT, Duncan ID (1998) Generation of oligodendroglial progenitors from neural stem cells. J Neurocytol 27: 475–489. 11246488 10.1023/a:1006953023845

[19] Avellana-Adalid V, Nait-Oumesmar B, Lachapelle F, Baron-Van Evercooren A (1996) Expansion of rat oligodendrocyte progenitors into proliferative “oligospheres” that retain differentiation potential. J Neurosci Res 45: 558–570. 8875321 10.1002/(SICI)1097-4547(19960901)45:5<558::AID-JNR6>3.0.CO;2-B

[20] Bain G, Kitchens D, Yao M, Huettner JE, Gottlieb DI (1995) Embryonic stem cells express neuronal properties in vitro. Dev Biol 168: 342–357. 7729574 10.1006/dbio.1995.1085

[21] Okabe S, Forsberg-Nilsson K, Spiro AC, Segal M, McKay RD (1996) Development of neuronal precursor cells and functional postmitotic neurons from embryonic stem cells in vitro. Mech Dev 59: 89–102. 8892235 10.1016/0925-4773(96)00572-2

[22] Brustle O, Jones KN, Learish RD, Karram K, Choudhary K, et al (1999) Embryonic stem cell-derived glial precursors: a source of myelinating transplants. Science 285: 754–756. 10427001 10.1126/science.285.5428.754

[23] Evans M (2011) Discovering pluripotency: 30 years of mouse embryonic stem cells. Nat Rev Mol Cell Biol 12: 680–686. 21941277 10.1038/nrm3190

[24] Zhou Q, Choi G, Anderson DJ (2001) The bHLH transcription factor Olig2 promotes oligodendrocyte differentiation in collaboration with Nkx2.2. Neuron 31: 791–807. 11567617 10.1016/s0896-6273(01)00414-7

[25] Du ZW, Li XJ, Nguyen GD, Zhang SC (2006) Induced expression of Olig2 is sufficient for oligodendrocyte specification but not for motoneuron specification and astrocyte repression. Molecular and Cellular Neuroscience 33: 371–380. 17035043 10.1016/j.mcn.2006.08.007

[26] Sharp J, Frame J, Siegenthaler M, Nistor G, Keirstead HS (2010) Human embryonic stem cell-derived oligodendrocyte progenitor cell transplants improve recovery after cervical spinal cord injury. Stem Cells 28: 152–163. 19877167 10.1002/stem.245 PMC3445430

[27] Zhang SC, Wernig M, Duncan ID, Brustle O, Thomson JA (2001) In vitro differentiation of transplantable neural precursors from human embryonic stem cells. Nat Biotechnol 19: 1129–1133. 11731781 10.1038/nbt1201-1129

[28] Ushio Y, Posner R, Posner JB, Shapiro WR (1977) Experimental spinal cord compression by epidural neoplasm. Neurology 27: 422–429. 558545 10.1212/wnl.27.5.422

[29] Delattre JY, Rosenblum MK, Thaler HT, Mandell L, Shapiro WR, et al (1988) A model of radiation myelopathy in the rat. Pathology, regional capillary permeability changes and treatment with dexamethasone. Brain 111 ( Pt 6): 1319–1336. 10.1093/brain/111.6.1319 3208060

[30] Talbott JF, Cao Q, Enzmann GU, Benton RL, Achim V, et al (2006) Schwann cell-like differentiation by adult oligodendrocyte precursor cells following engraftment into the demyelinated spinal cord is BMP-dependent. Glia 54: 147–159. 16921543 10.1002/glia.20369 PMC2813493

[31] Cao Q, He Q, Wang Y, Cheng X, Howard RM, et al. Transplantation of ciliary neurotrophic factor-expressing adult oligodendrocyte precursor cells promotes remyelination and functional recovery after spinal cord injury. J Neurosci 30: 2989–3001. 10.1523/JNEUROSCI.3174-09.2010 PMC2836860 20181596

[32] Hofstetter CP, Holmstrom NA, Lilja JA, Schweinhardt P, Hao J, et al (2005) Allodynia limits the usefulness of intraspinal neural stem cell grafts; directed differentiation improves outcome. Nat Neurosci 8: 346–353. 15711542 10.1038/nn1405

[33] Iwashita Y, Fawcett JW, Crang AJ, Franklin RJ, Blakemore WF (2000) Schwann cells transplanted into normal and X-irradiated adult white matter do not migrate extensively and show poor long-term survival. Exp Neurol 164: 292–302. 10915568 10.1006/exnr.2000.7440

[34] Keirstead HS, Nistor G, Bernal G, Totoiu M, Cloutier F, et al (2005) Human embryonic stem cell-derived oligodendrocyte progenitor cell transplants remyelinate and restore locomotion after spinal cord injury. J Neurosci 25: 4694–4705. 15888645 10.1523/JNEUROSCI.0311-05.2005 PMC6724772

[35] Cao Q, He Q, Wang Y, Cheng X, Howard RM, et al (2010) Transplantation of ciliary neurotrophic factor-expressing adult oligodendrocyte precursor cells promotes remyelination and functional recovery after spinal cord injury. J Neurosci 30: 2989–3001. 20181596 10.1523/JNEUROSCI.3174-09.2010 PMC2836860

[36] Billon N, Jolicoeur C, Ying QL, Smith A, Raff M (2002) Normal timing of oligodendrocyte development from genetically engineered, lineage-selectable mouse ES cells. J Cell Sci 115: 3657–3665. 12186951 10.1242/jcs.00049

[37] Franklin RJ, Bayley SA, Blakemore WF (1996) Transplanted CG4 cells (an oligodendrocyte progenitor cell line) survive, migrate, and contribute to repair of areas of demyelination in X-irradiated and damaged spinal cord but not in normal spinal cord. Exp Neurol 137: 263–276. 8635541 10.1006/exnr.1996.0025

[38] Jeffery ND, Crang AJ, O’Leary M T, Hodge SJ, Blakemore WF (1999) Behavioural consequences of oligodendrocyte progenitor cell transplantation into experimental demyelinating lesions in the rat spinal cord. Eur J Neurosci 11: 1508–1514. 10215903 10.1046/j.1460-9568.1999.00564.x

[39] Schultheiss TE, Stephens LC (1992) Invited review: permanent radiation myelopathy. Br J Radiol 65: 737–753. 1393407 10.1259/0007-1285-65-777-737

[40] Nave KA, Trapp BD (2008) Axon-glial signaling and the glial support of axon function. Annu Rev Neurosci 31: 535–561. 18558866 10.1146/annurev.neuro.30.051606.094309

[41] Trapp BD, Nave KA (2008) Multiple sclerosis: an immune or neurodegenerative disorder? Annu Rev Neurosci 31: 247–269. 18558855 10.1146/annurev.neuro.30.051606.094313

[42] Cummings BJ, Uchida N, Tamaki SJ, Salazar DL, Hooshmand M, et al (2005) Human neural stem cells differentiate and promote locomotor recovery in spinal cord-injured mice. Proc Natl Acad Sci U S A 102: 14069–14074. 16172374 10.1073/pnas.0507063102 PMC1216836

[43] Karimi-Abdolrezaee S, Eftekharpour E, Fehlings MG (2004) Temporal and spatial patterns of Kv1.1 and Kv1.2 protein and gene expression in spinal cord white matter after acute and chronic spinal cord injury in rats: implications for axonal pathophysiology after neurotrauma. Eur J Neurosci 19: 577–589. 14984408 10.1111/j.0953-816x.2004.03164.x

[44] Mitsui T, Shumsky JS, Lepore AC, Murray M, Fischer I (2005) Transplantation of neuronal and glial restricted precursors into contused spinal cord improves bladder and motor functions, decreases thermal hypersensitivity, and modifies intraspinal circuitry. J Neurosci 25: 9624–9636. 16237167 10.1523/JNEUROSCI.2175-05.2005 PMC6725721

[45] Cao Q, Zhang YP, Iannotti C, DeVries WH, Xu XM, et al (2005) Functional and electrophysiological changes after graded traumatic spinal cord injury in adult rat. Exp Neurol 191 Suppl 1S3–S16. 15629760 10.1016/j.expneurol.2004.08.026

[46] Bruce CC, Zhao C, Franklin RJ (2010) Remyelination - An effective means of neuroprotection. Horm Behav 57: 56–62. 19538961 10.1016/j.yhbeh.2009.06.004

[47] Jadasz JJ, Aigner L, Rivera FJ, Kury P (2012) The remyelination Philosopher’s Stone: stem and progenitor cell therapies for multiple sclerosis. Cell Tissue Res. 10.1007/s00441-012-1331-x 22322424

[48] Goldberg JL, Barres BA (2000) The relationship between neuronal survival and regeneration. Annu Rev Neurosci 23: 579–612. 10845076 10.1146/annurev.neuro.23.1.579

[49] Wilkins A, Majed H, Layfield R, Compston A, Chandran S (2003) Oligodendrocytes promote neuronal survival and axonal length by distinct intracellular mechanisms: a novel role for oligodendrocyte-derived glial cell line-derived neurotrophic factor. J Neurosci 23: 4967–4974. 12832519 10.1523/JNEUROSCI.23-12-04967.2003 PMC6741206

[50] Du Y, Dreyfus CF (2002) Oligodendrocytes as providers of growth factors. Journal of Neuroscience Research 68: 647–654. 12111826 10.1002/jnr.10245

[51] Dai X, Lercher LD, Clinton PM, Du Y, Livingston DL, et al (2003) The trophic role of oligodendrocytes in the basal forebrain. J Neurosci 23: 5846–5853. 12843289 10.1523/JNEUROSCI.23-13-05846.2003 PMC6741226

[52] Maier IC, Schwab ME (2006) Sprouting, regeneration and circuit formation in the injured spinal cord: factors and activity. Philos Trans R Soc Lond B Biol Sci 361: 1611–1634. 16939978 10.1098/rstb.2006.1890 PMC1664674

